# A long-term retrospective cohort-based risk-benefit analysis of augmenting total cumulative I-131 activity to 37GBq in differentiated thyroid cancer patients with skeletal metastases

**DOI:** 10.1371/journal.pone.0294343

**Published:** 2023-11-14

**Authors:** Sivasankar Kanankulam Velliangiri, Sanjana Ballal, Madhav Prasad Yadhav, Madhavi Tripathi, Swayamjeet Satapathy, Chandrasekhar Bal

**Affiliations:** Department of Nuclear Medicine, All India Institute of Medical Sciences, New Delhi, India; Emory University, UNITED STATES

## Abstract

**Objective:**

Skeletal metastases in differentiated thyroid cancer (DTC) patients are associated with poor prognosis. The objective was to determine the maximum I-131 cumulative activity that could be safely administered without compromising efficacy. The secondary objective was to identify other prognostic factors affecting survival outcomes.

**Materials and methods:**

This was a retrospective cohort study done at a tertiary-care institution comprising of data from January 1990-June 2020. 489 DTC patients having skeletal metastases with ≥12 months follow-up were included. Ninety-six percent of patients had thyroidectomy followed by radioiodine therapy for skeletal metastases. All patients were on oral suppressive levothyroxine tablets. External beam radiotherapy (EBRT) and oral tyrosine kinase inhibitors were used whenever indicated. The main outcome measures were overall survival (OS), progression-free survival (PFS), and adverse-events.

**Results:**

There were 347 (71%) females and 324 (66%) had follicular carcinoma thyroid. Median follow-up was 78 (interquartile range, IQR: 37–153) months. 333 patients (68%) received ≤37GBq I-131 cumulative activity (group 1) and 156 patients (32%) received >37GBq cumulative RAI activity (group 2). Overall median OS and PFS were 74 (95% confidence interval (CI): 62.2–85.8) and 48 (95%CI: 40.5–55.4) months, respectively. The 5-, 10-, 15- and 20-year estimated overall survival probabilities were 55.7%, 28.4%, 14% and 8.3%, respectively. On multivariate analysis, age(<55years) (p<0.001), female gender(p = 0.01), cumulative I-131 activity >37GBq (p<0.001) and EBRT(p = 0.001) were favourably associated with OS; no factors were significantly associated with PFS. The median OS for groups 1 & 2 were 51 versus 90 months (p<0.001) & median PFS for groups 1 & 2 were 45 versus 53 months respectively (p = 0.9). However, cumulative activity >37GBq resulted in more adverse events (2.4%), particularly bone marrow suppression (3.5%).

**Conclusion:**

For better survival outcomes, cumulative I-131 activity upto 37GBq could be administered with acceptable toxicity to DTC patients with skeletal metastases.

## Introduction

Differentiated thyroid cancer (DTC) is one of the most common endocrine malignancies with an annual incidence of 14.3, and a death rate of 0.5 per 100,000 population in the United States [1]. The incidence is increasing in India as well, especially in people younger than 40 years age [2]. DTC is indolent in nature, and about 95% of patients present with intrathyroidal or locoregional disease. However, depending on socioeconomic status [3] of the country, distant metastasis at presentation varies from 3% - 15% [4–8]. The common sites of metastases include lungs and bone [9]. The bone metastasis severely affects the quality of life with reported mean survival of 4 years [10]. The differentiated thyroid cancer cells, even in distant metastatic sites, express sodium iodide symporter that facilitates radioiodine uptake [11]. The treatment of distant metastasis with I-131 remains one of the most non-controversial issues in the management of DTC [12–14]. However, the maximum activity of I-131 to be administered remains controversial [15]. The initial excellent I-131 uptake in skeletal metastases eventually may end up in the radioiodine refractory disease (RR-DTC) [16]. Once the tumour becomes RR-DTC, treatment with radioiodine is no longer recommended [13]. Other treatment options include surgical excision, if feasible; external beam radiotherapy (EBRT) mostly as palliative option, and bone protective agents to avoid skeletal-related events (SREs). The newer treatment options approved by Food and Drug Administration for RR-DTC include tyrosine kinase inhibitors (TKIs), namely Sorafenib, Lenvatinib and recently, Cabozantinib [10, 12, 13, 17].

Various studies have attributed different prognostic factors for survival outcomes and these studies were limited by sample size [18–21]. Therefore, the main objective was to determine the maximum cumulative activity of I-131 that could be safely administered without compromising efficacy in a large cohort of patients treated uniformly by a standard protocol and meticulously followed in a single centre. The secondary objective was to identify other prognostic factors affecting survival outcomes in these DTC patients with skeletal metastases.

## Materials and methods

Electronic as well as physical database of patients who have been treated with I-131 for bone metastasis in DTC and were followed up in thyroid clinic from January 1990 to June 2020 were retrieved during January 2022 to April 2022 and the data sheet included deidentified patient data. Patients with age ≥ 18 years, biopsy-proven primary DTC, bone metastases demonstrated on whole-body radioiodine scan and/or any other imaging modalities and received radioiodine therapy for metastases with a minimum follow-up period of 12 months (or expired within 12 months) were included in the study. Patients in whom the clinical evaluation/documentation were inadequate or follow-up <12 months were excluded from the analysis. The patients were divided into two groups based on cumulative I-131 activity ≤37GBq (group-1) and >37GBq (group-2). The institutional ethics committee waived-off the informed consent in view of retrospective study without disclosing the patient’s identity (IECPG-720/25.11.2021, RT-36/23.12.2021).

### Diagnosis and treatment protocol

Baseline biochemical investigations like serum thyroglobulin (Tg), Anti-thyroglobulin antibody (ATg) and thyroid stimulating hormone (TSH) levels were done in all patients. As per institutional policy, all patients underwent TSH-stimulated [either levothyroxine withdrawal for 3–4 weeks or after recombinant TSH (rhTSH) stimulation] I-131whole-body scan (Dx-WBS). The Dx-WBS was performed at 24–48 hours after administration of ~74MBq I-131. After giving radiation safety instructions, 7.4GBq I-131 were administered to patients with skeletal metastases. The patients were admitted to the isolation ward until the radiation level drops below 50μSv/h(5mR/h) at 1-meter distance as per national discharge policy. Then post-therapy I-131 whole-body scans (PTS) were performed in all patients at 72 hours to assess any additional sites of disease. Subsequently, the patients were started on 2μg/kg body weight levothyroxine orally daily and thyroxine doses adjusted keeping TSH levels between 0.01–0.1 μIU/ml.

### Follow-up

If patients had significant disease noted in the post-therapy scan, second dose of I-131 was administered and post-therapy scan was done to document the response. The serial radioiodine therapy was continued until patients showed complete response or developed progression or radioiodine refractory disease. Patients were followed up six monthly once they became disease-free for the first five years and annually thereafter. During follow-up, a target TSH level of ≤0.1uIU/ml was maintained and adverse events were monitored by clinical examination and biochemical investigations(CBC, renal function test and liver function test)

Once the patient achieved remission, subsequent follow-ups were based on physical examinations, Tg and ATg estimations, and if there was suspicion of recurrence, radiological investigations like X-ray, US, CT and/or ^18^F-FDG PET/CT were performed to look for non-iodine concentrating structural disease.

In cases of oligometastatic diseases, and cases of impending spinal cord compression, patients were referred for external beam radiotherapy (EBRT). In cases of RR-DTC, redifferentiation therapy earlier or recently tyrosine kinase inhibitors (TKIs) were advised [22, 23].

### End points

The primary endpoint was Overall survival (OS) and the secondary endpoints were progression free survival (PFS), remission rate, recurrence rate and RR-DTC rate.

### Definitions

Remission was defined as Tg (stimulated) ≤10ng/ml [13] with negative ATg and Dx-WBS. Recurrence was defined as the appearance of new lesion in any imaging modalities after the patient has achieved documented remission. Progression was defined as two-fold increase in Tg levels from baseline if ATg is negative, increasing trend of ATg if ATg is positive and/or appearance of any new lesion in any of the imaging (WBS, CT, FDG PET/CT). Radioiodine refractory disease was defined as the 1. De novo non iodine concentrating lesions, 2. Some non-iodine concentrating lesions in initial WBS, 3. Previously iodine concentrating lesions becoming non concentrating and confirmed by imaging modalities, 4. DTC metastasis progression despite radioiodine uptake 5. DTC metastasis progression despite a cumulative activity of >22.2GBq [15]. PFS was calculated from the date of radioiodine therapy to date of progression. OS was calculated from the time of radioiodine therapy to time of death. We have classified all deaths as thyroid cancer-related deaths in order to present our data under the most conservative and worst-case scenario.

### Statistical analysis

Qualitative data were expressed as numbers and percentages. Difference between groups were assessed using Chi-square or Fisher exact test. The normality of the data was checked by Kolmogorov–Smirnov test. Normal data were expressed as mean (±SD); skewed data were expressed as median and inter-quartile range (±IQR). PFS and OS were estimated by Kaplan-Meier survival analysis and compared between groups using log-rank test. Univariate and multivariate analyses were done using cox proportional hazard regression model. P-value <0.05 was considered significant. Statistical analyses were performed using Medcalc and SPSS version 21.

## Results

**Fig 1:**
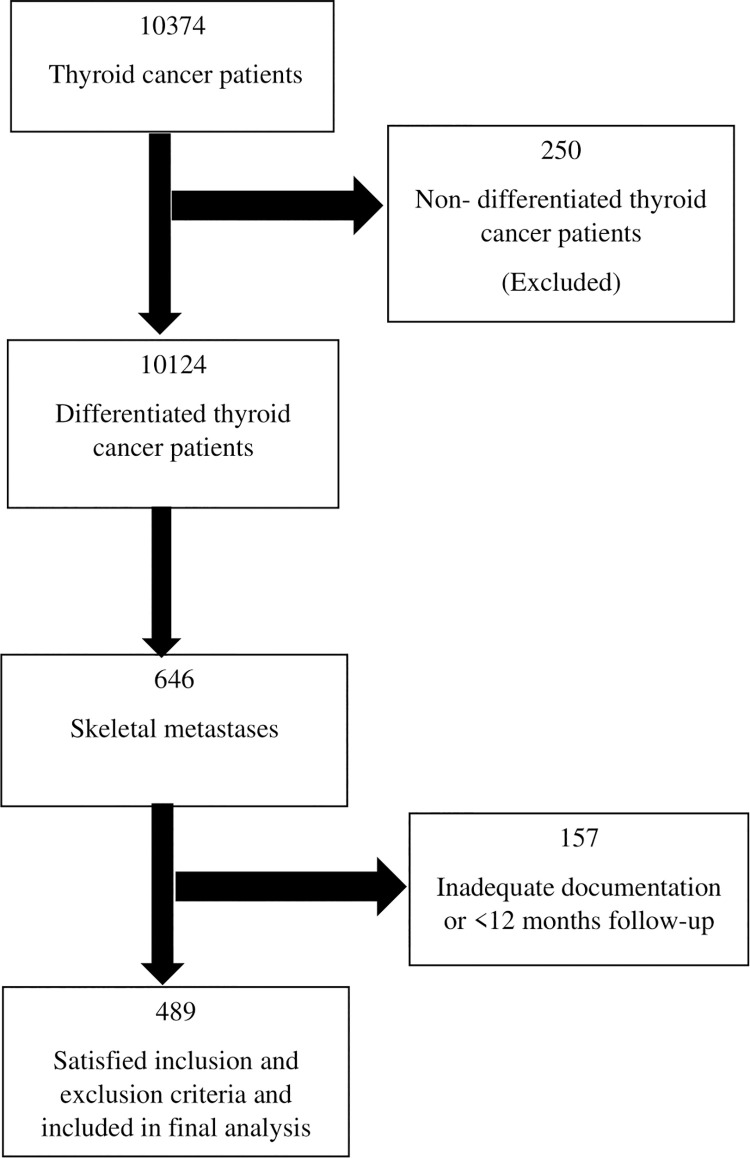
Study design.

Out of 10,374 thyroid cancer patients registered, 10,124 had DTC and 646 patients had skeletal metastases. Of the 646 patients with skeletal metastases, 489 patients were included in the final analysis (Fig 1). The prevalence of skeletal metastases among thyroid cancer patients in our study was 6.4%.

### Clinical characteristics

The mean age of the patients was 52.4 ± 11.9 years and equally distributed between cut-off age of 55 years:258 patients(52.8%) were <55 years and 231 patients (47.2%) were ≥55 years of age at the time of diagnosis. There were 347 female patients (71%) and 142 male patients (29%). The skeletal metastasis were diagnosed at different time points as follows: 261 (53.3%) patients presented with skeletal metastasis, 158 (32.3%) patients had occult metastasis detected on initial pre- therapy diagnostic I-131 WBS, 19(3.9%) patients were diagnosed with skeletal metastasis on initial post therapy I-131 WBS, and 51 (10.5%) patients were diagnosed to have skeletal metastases during subsequent follow-up (Table 1).

**Table 1:** Patient characteristics at the time of presentation.

S.no	Parameter		N(percentage)
1	Age	<55 years	258(52.8)
		≥55 years	231(47.2)
2	Sex	Female	347(71)
		Male	142(29)
3	Preoperative metabolic status	Euthyroid	466(95.3)
		Hypothyroid	12(2.5)
		Hyperthyroid	11(2.2)
4	Initial presentation	Solitary thyroid nodule	194(39.7)
		MNG	34(7)
		Metastases	261(53.3)
5	Metastases detection in patients with STN & MNG	Diagnostic WBS	158(69.3)
		Post-therapy WBS	19(8.3)
		Subsequent follow-up	51(22.4)
6	Surgery	TT/NTT/STT	399(81.6)
		HT	74(15.1)
		Nodulectomy	8(1.6)
		Inoperable	8(1.6)
7	Nodal dissection	Done	117(23.9)
		Not done	340(69.5)
		Not mentioned in HPE	32(6.5)
8	Histopathology	Papillary	165(33.7)
		Follicular carcinoma	324(66.3)
9	EBRT	Given	182(37.2)
		Not given	307(62.8)
10	TKIs	Given	54(11)
		Not given	435(89)
11	Method of preparation for radioiodine therapy	RhTSH	28(5.7)
		Levothyroxine withdrawal	461(94.3)
12	I-131 cumulative dose	≤ 37GBq	333(68.1)
		>37GBq	156(31.9)
13	Status after initial treatment	Remission	136(27.8)
		Progression	280(57.3)
		Persistent disease	73(14.9)
14	Outcome at the time of analysis	Lost to follow-up	37(7.5)
		Death	254(52)
		Remission	78(16)
		Persistent/progression	120(24.5)
15	Second Malignancy		10(2)
16	Adverse events		20(4)
17	Recurrence		58(11.8)
	Recurrence regions	Skeletal	34(58.7)
		Lungs	7(12)
		Lymph nodes	6(10.4)
		Lungs with skeletal	5(8.7)
		Local recurrence	4(6.8)
		Skin	1(1.7)
		Brain	1(1.7)

MNG-multinodular goiter; TT-total thyroidectomy; NTT-near-total thyroidectomy; STT-subtotal thyroidectomy; HT-hemithyroidectomy; HPE-histopathological examination; rhTSH- recombinant TSH; EBRT- External beam radiotherapy; TKIs- tyrosine kinase inhibitors.

### Treatment characteristics

Majority of patients (81.6%) underwent total or near-total thyroidectomy. A total of 117 patients (23.9%) had neck lymph node dissection along with thyroidectomy. On postoperative histopathological examination, 324 patients (66.3%) had follicular carcinoma and 165 patients (33.7%) had papillary carcinoma. Preparation for radioiodine administration was done using conventional levothyroxine withdrawal in majority of the patients (461 patients, 94.3%) and a minority with recombinant TSH (rhTSH) as off-label use (28 patients, 5.7%) (Table 1). The median cumulative activity of I-131 administered was 29.6GBq (IQR 18.5–44.2GBq). Of the patients who received radioiodine, 333 patients (68%) received ≤37GBq I-131cumulative activity(group-1), 156 patients (32%) received >37GBq cumulative RAI activity(group-2). The baseline characteristics between 2 groups are given in Table 2. The median cumulative activity for group 1 and group 2 were 22.2GBq (IQR-14.8–29.6 GBq) and 49.6GBq (IQR- 44.4–57.4GBq) respectively. A total of 182 patients (37.2%) had received external beam radiotherapy, and 54 patients (11%) has received TKIs like sorafenib and/or Lenvatinib. Also,15 patients (3.1%) had received redifferentiation therapy before TKIs were available in India (Table 1).

**Table 2:** Comparison of baseline characteristics between 2 groups.

S.No	Parameter		Group -1 (I-31≤37GBq)	Group-2 (I-131>37GBq)	P—value
1	Age	<55 years	170	88	0.27
		≥55 years	163	68	
2	Sex	Female	239	108	0.56
		Male	94	48	
3	Preoperative metabolic status	Euthyroid	320	146	0.47
		Hypothyroid	7	5	
		Hyperthyroid	6	5	
4	Initial presentation	Solitary thyroid nodule	139	55	0.32
		MNG	24	10	
		Metastases	170	91	
5	Metastases detection in patients with STN & MNG	Diagnostic WBS	112	46	0.07
		Post-therapy WBS	10	9	
		Subsequent follow-up	41	10	
6	Surgery	TT/NTT/STT	266	133	0.22
		HT	57	17	
		Nodulectomy	4	4	
		Inoperable	6	2	
7	Nodal dissection	Done	84	33	0.46
		Not done	229	111	
		Not mentioned in HPE	20	12	
8	Histopathology	Papillary carcinoma	116	49	0.46
		Follicular carcinoma	217	107	
9	Method of preparation for radioiodine therapy	rhTSH	18	10	0.66
		Levothyroxine withdrawal	315	146	

MNG-multinodular goiter; TT-total thyroidectomy; NTT-near-total thyroidectomy; STT-subtotal thyroidectomy; HT-hemithyroidectomy; HPE-histopathological examination; rhTSH- recombinant TSH.

### Treatment outcomes

The median follow-up in our study population was 78 (IQR: 37–153) months. After initial treatment, 136 patients (27.8%) achieved remission. Out of 136 patients with initial remission, subsequently 58(42.6%) patients developed recurrent disease, with the median time to recurrence of 60 (IQR: 24–102) months. Majority of recurrences were noted in new skeletal sites in 34 (58.7%) patients. Interestingly, RR-DTC developed in 339 patients (69.3%) at the end of follow-up period (S1 Table). At the time of analysis, 254 (52%) patients expired, 120 (24.5%) patients had progressive/persistent disease, 78 (16%) patients were in remission, and 37 (7.5%) patients were lost to follow-up (Table 1).

### Overall survival and prognostic factors

The median OS was 74 (95%CI: 62.2–85.8) months in our study population. The 5-, 10-, 15- and 20-year estimated survival probabilities were 55.7%, 28.4%, 14% and 8.3%, respectively. The median OS for ≤37GBq group and >37GBq group were 51(95%CI: 41.6–60.3) and 90 (95%CI: 77.9–102) months (p<0.001), respectively.

**Fig 2:**
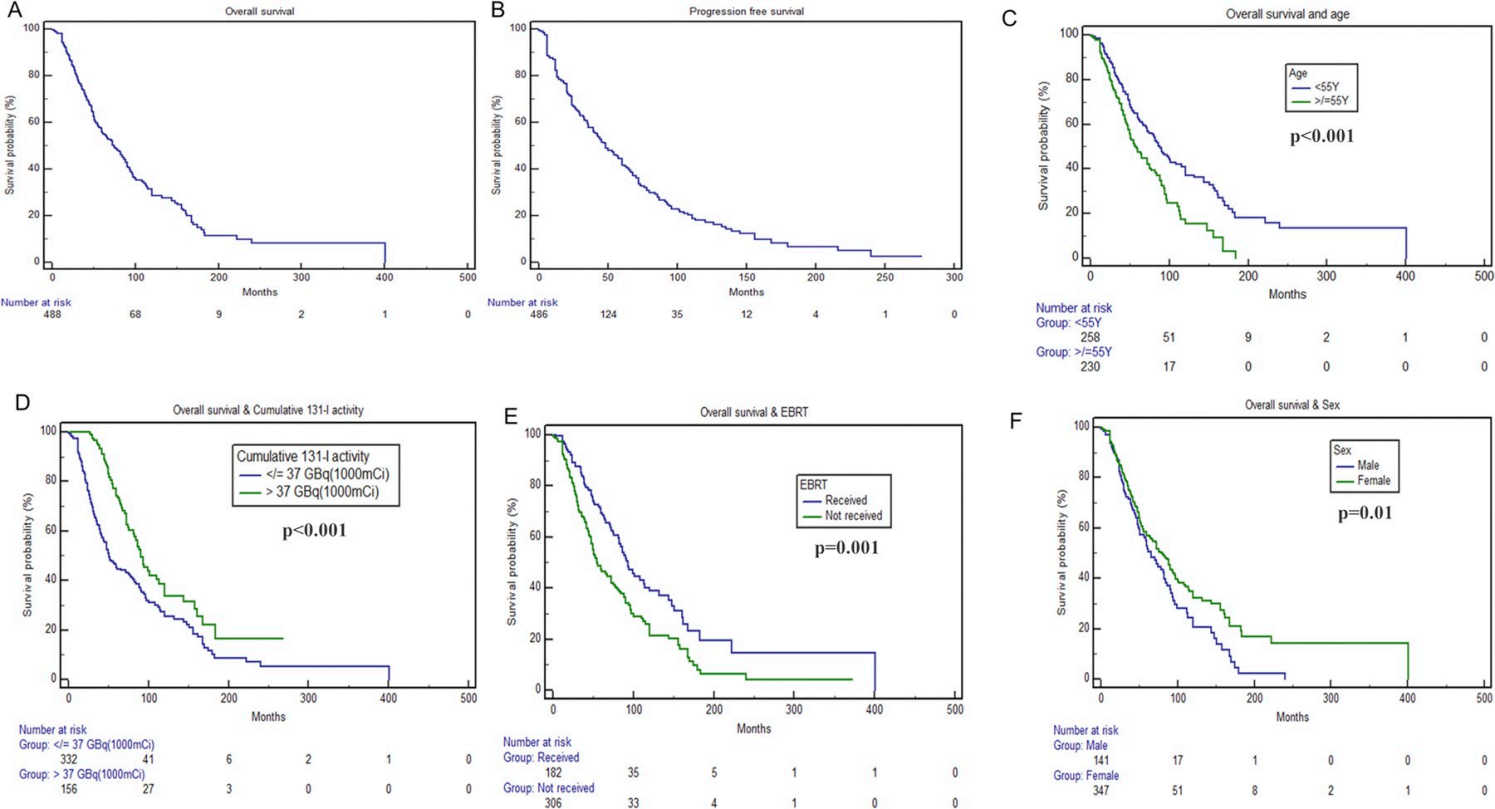
Kaplan Meier survival curves.

For univariate analysis, age, sex, histopathology, extrathyroidal extension, N stage, metastases presentation, cumulative I-131 activity and EBRT were included. Age, gender, cumulative I-131 activity, and EBRT were found to significantly impact OS. On multivariate analysis, male gender (p = 0.01; HR-1.38; 95%CI: 1.07–1.80) and cumulative I-131 activity ≤37GBq (p = <0.001; HR-1.84; 95%CI- 1.39–2.44) had adverse prognostic effects; however, age <55years (p = <0.001; HR- 0.61; 95%CI: 0.47–0.79), and those who received EBRT (p = 0.001; HR-0.64; 95%CI:0.49–0.83) had favourable effects on OS. (Table 3 and Fig 2).

**Table 3:** Univariate and multivariate cox proportional hazards regression analysis of prognostic factors affecting overall survival.

S.No	Parameter		Univariate Analysis		Multivariate analysis	
			P value	Hazards Ratio (95% Confidence interval)	P value	Hazards Ratio (95% Confidence interval)
1	Age	<55 Years	**<0.001**	0.59(0.46–0.76)	**<0.001**	0.61(0.47–0.79)
		≥ 55 years				
2	Sex	Male	**0.02**	1.35(1.04–1.75)	**0.01**	1.38(1.07–1.80)
		Female				
3	Histopathology	Papillary	0.87	0.98(0.75–1.27)		
		Follicular				
4	Extrathyroidal extension	Yes	0.39	1.38(0.66–2.88)		
		No				
5	N stage	N0	0.678	1.07(0.77–1.48)		
		N1				
6	Metastases presentation	Only skeletal metastases	0.244	0.83(0.60–1.14)		
		Multiorgan metastases				
7	Cumulative I-131 Dose	≤37GBq	**<0.001**	2.03(1.53–2.68)	**<0.001**	1.84(1.39–2.44)
		>37GBq				
8	EBRT	Yes	**<0.001**	0.58(0.45–0.76)	**0.001**	0.64(0.49–0.83)
		No				

EBRT-external beam radiotherapy; P<0.05 is considered significant. GBq-Gigabecquerel.

A-Overall survival. B- Progression free survival. C- Kaplan Meier survival curves for overall survival and age. D- Kaplan Meier survival curves for overall survival and different cumulative I-131 activity. E- Kaplan Meier survival curves for overall survival and EBRT; F- Kaplan Meier survival curves for overall survival and sex. N.B: Number at risk at baseline for OS was 488 because 1 patient died at baseline. Number at risk for PFS was 486 at baseline because 1 patient died at baseline and 2 other patients did not undergo biochemical and radiological tests to assess for progression.

### Progression free survival and prognostic factors

The median PFS in our study was 48 (95%CI:40.5–55.4) months. The median PFS for ≤37GBq group and >37GBq group were 45(95%CI: 34.7–55.2) and 53 (95%CI: 43.5–62.4) months(p = 0.9), respectively (Fig 2). Interestingly, none of the above-mentioned factors was found to significantly affect PFS.

### Adverse events

Adverse events were noted in 20(4%) patients. Bone marrow suppression was noted in 17 (3.5%) patients and 14 among them received >37GBq radioiodine (p<0.001) (Table 4). Second primary malignancy (SPM) was recorded in 10 patients (2%) (Table 5. There was no significant difference in the incidence of second malignancy among different cumulative activity groups (p = 0.18).

**Table 4:** Adverse events.

S.no	Age (Year)	Sex	Adverse Event	TIME (Month)	HPE	131-I Cumulative activity GBq	EBRT
1	51	F	Bone Marrow Suppression	28	Follicular	32.38	N
2	18	F	Ovarian failure	156	Papillary	46.06	Y
3	52	M	Bone Marrow Suppression	38	Papillary	50.5	N
4	36	F	T-MDS	145	Papillary	38.11	Y
5	42	F	Bone Marrow Suppression	52	Papillary	48.1	Y
6	45	F	Bone Marrow Suppression	128	Follicular	53.6	Y
7	62	M	Bone Marrow Suppression	113	Papillary	41.07	Y
8	47	M	Bone Marrow Suppression	106	Follicular	66.6	Y
9	68	M	Bone Marrow Suppression	60	Follicular	44.4	Y
10	57	F	Bone Marrow Suppression	60	Follicular	59.2	Y
11	48	F	Bone Marrow Suppression	65	Papillary	51.8	N
12	52	F	Bone Marrow Suppression	72	Follicular	57.35	Y
13	55	F	Bone Marrow Suppression	97	Follicular	48.1	N
14	60	F	Bone Marrow Suppression	56	Follicular	51.8	N
15	41	F	Bone Marrow Suppression	56	Follicular	59.2	N
16	50	F	Bone Marrow Suppression	59	Papillary	37.15	Y
17	50	F	Bone Marrow Suppression	29	Follicular	37	Y
18	58	F	Bone Marrow Suppression	32	Follicular	29.6	Y
19	65	F	Bone Marrow Suppression	105	Follicular	32.56	Y
20	65	F	Died next day after radioiodine therapy	Immediate	Follicular	33.3	N

M-male; F- Female; t-MDS- treatment-related myelodysplastic syndrome; HPE- histopathological examination; EBRT-external beam radiotherapy; Y-yes; N-no. GBq-Gigabecquerel.

**Table 5:** Second malignancy.

S.no	Age (years)	Sex	Diagnosis	Time After diagnosis (Month)	HPE	Cumulative 131-I activity GBq	EBRT	TKI
1	67	M	Carcinoma Prostate	24	Follicular	32	N	N
2	59	M	Leiomyosarcoma	32	Follicular	22	N	N
3	60	M	Carcinoma esophagus	20	Papillary	16.6	N	N
4	36	F	t-MDS	144	Papillary	38.1	Y	Y
5	59	M	Carcinoma urinary bladder	156	Papillary	31.5	Y	N
6	58	F	Meningioma	synchronous	Follicular	37	Y	N
7	58	F	Carcinoma breast	18	Follicular	29.6	Y	N
8	78	F	Carcinoma breast	synchronous	Follicular	29.6	Y	N
9	60	F	Carcinoma cervix	synchronous	Follicular	29.6	Y	N
10	58	F	Renal cell carcinoma	synchronous	Papillary	22	Y	Y

M-male; F- Female; t-MDS- treatment-related myelodysplastic syndrome; HPE- histopathological examination; EBRT-external beam radiotherapy; TKIs- tyrosine kinase inhibitors; Y-yes; N-no. GBq-Gigabecquerel.

## Discussion

This study represents more than three decades of experience in treating thyroid cancer patients. Though other studies on this subject are available, they are of limited sample size or with limited duration of follow-up. Prospective studies are beyond the scope of analysis in follow-up of slow growing thyroid cancers. Though current study is retrospective, it has the advantage of evaluating outcomes in a real-world setting. As per our knowledge, 489 DTC patients with skeletal metastasis from a single institution with median follow-up of 78 months constitutes the largest series that is published until now.

We observed 6.4% skeletal metastases from DTC in this series that is slightly higher than the prevalence reported from other studies [18, 24–26]. Contrary to western data, publication from another institution from India, albeit in small number of patients, showed even higher prevalence (28/140 patients; 15.7%) of skeletal metastases [27]. This probably, could be explained due to late presentation that is unique to oncologic presentations in low- and middle-income countries (LMICs).

A total 136 (27.8%) patients achieved complete remission; however, 58/136 (42.6%) patients developed disease recurrence with median time to recurrence of 60 months. Thus, these patients need life-long follow-up. This large single centre study shows that DTC patients with skeletal metastases have comparatively fewer chances of long-term remission (only 16% in this series).

At the time of analysis, >50% of the patients were deceased (Table 1). However, our median OS (74 vs 57 months) and 10-year survival probability (28.4% vs 15.3%) were higher than that reported by Jannin et al. in a recent publication from France [19]. These findings could be attributed to increased median cumulative I-131 activity administered to our patients (29.6 vs 11.1GBq).

The maximum cumulative activity of I-131 that can be administered for treatment is still a question that needs to be answered. Current practice guidelines recommend administration of maximum up to 22GBq of I-131 in view of adverse effects and possibility of developing second primary malignancy [13]. On contrary, the median cumulative activity in our study was 29.6GBq. Various studies reported in the literature have varied cumulative activities with investigator’s choice as low as 11.1GBq [6, 19, 20, 28]. Previous studies have shown that cumulative activity of radioiodine [21, 29] plays an important role in survival which was again confirmed by this large cohort study. We observed that patients who received cumulative I-131 activity of >37GBq had better overall survival.

The bone marrow suppression noted in our study was 3.5% and majority of them received >37GBq cumulative activity. The incidence of bone marrow suppression was higher than those reported by Edmonds et al., (1.1%) [30] and Grunwald et al., (1.4%) [31] whereas it was lower than that reported by Alexander et al (4.4%) [32]. The second primary malignancy in our study was 2% which is higher than that reported by Mei et al., (0.9%) and lesser than that reported by Brown et al., (~7%) [33, 34]. The reason for the higher incidence of bone marrow suppression and second primary malignancy may be because this study population includes only skeletal metastases patients who actually received higher doses of I-131 whereas other studies included all patients with DTC treated with I-131. Though the maximum cumulative activity of 22.2-37GBq is debatable, our study provides evidence for safely raising the ceiling of cumulative radioiodine activity up to 37GBq without significantly increasing the adverse effects of radioiodine, especially in LMICs where availability of alternative treatment options like TKIs is limited and expensive.

Interestingly, at the end of follow-up, 339(69.3%) patients were radioiodine refractory. Earlier studies [6, 19, 26, 35, 36] showed that radioiodine refractory thyroid cancer is associated with poor outcomes. Thus, we advocate that newer treatment options like TKIs should be offered in radioiodine refractory thyroid cancer patients.

The study suffers all the limitations of the retrospective study design, e.g., incomplete documentation of progression in earlier era in some of the patients, selection bias of a tertiary referral centre, and individualized non-radioiodine treatment methods. However, we made all efforts to telephonically communicate with the patients/family members about the current health status of patients. Also, our study did not assess the quality of life with radioiodine therapy, social factors [37], prevalence and contribution of skeletal related events on the overall survival.

## Conclusion

Patients with skeletal metastases who received ≤37GBq radioiodine therapy had the poorer outcomes than those who received more than >37GBq. However, significant adverse events were noticed in patients who were administered >37GBq of I-131. Thus, a cumulative dose of up to 37GBq radioiodine can be safely administered for DTC patients with skeletal metastases.

## Supporting information

S1 File (XLSX)

S1 Table Number of patients in each category of radioiodine refractory-DTC. (PDF)
