# Supplementary material for: A long-term retrospective cohort-based risk-benefit analysis of augmenting total cumulative I-131 activity to 37GBq in differentiated thyroid cancer patients with skeletal metastases
Source: PLoS One. 2023 Nov 14;18(11):e0294343. doi: 10.1371/journal.pone.0294343 (PMC10645322; doi:10.1371/journal.pone.0294343)
Supplement: S1 Table — (PDF) [file pone.0294343.s002.pdf]

Supplementary table – 1: Number of patients in each category of Radioiodine refractory -DTC

S.No	Criteria	No. of patients (percentage)
1	De novo non-iodine concentrating lesions	9(2.6)
2	Presence of non-iodine concentrating lesions in the initial whole-body scan	6 (1.8)
3	Transition from iodine-concentrating lesions to non-concentrating lesions, as confirmed by imaging modalities	9(2.6)
4	Disease progression in DTC metastases despite radioiodine uptake	9(2.6)
5	Disease progression in DTC metastases despite a cumulative administered activity of >22.2 GBq	306 (90.4).

DTC- differentiated thyroid cancer.
